# ZnO Nanoparticles as Potent Inducers of Dermal Immunosuppression in Contact Hypersensitivity in Mice

**DOI:** 10.1021/acsnano.4c04270

**Published:** 2024-10-14

**Authors:** Shuyuan Wang, Marit Ilves, Kuunsäde Mäenpää, Lan Zhao, Hani El-Nezami, Piia Karisola, Harri Alenius

**Affiliations:** † School of Biological Sciences, University of Hong Kong, Pok Fu Lam Road, 999077 Hong Kong, People’s Republic of China; ‡ Human Microbiome Research Program, University of Helsinki, Haartmaninkatu 3, 00290 Helsinki, Finland; § School of Medicine, Institute of Public Health and Clinical Nutrition, University of Eastern Finland, P.O. Box 1627, 70211 Kuopio, Finland; ∥ Institute of Environmental Medicine (IMM), Karolinska Institutet, 171 77 Stockholm, Sweden

**Keywords:** contact hypersensitivity (CHS), mouse model, metal oxide nanoparticles, RSEQ, inflammation, chemotaxis

## Abstract

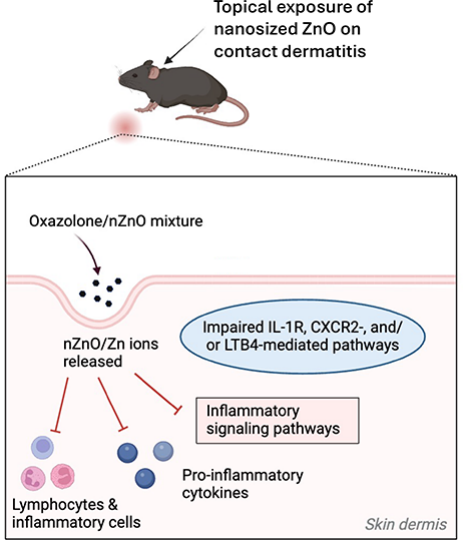

Nanosized zinc oxide (nZnO) metal particles are used in skin creams and sunscreens to enhance their texture and optical properties as UV filters. Despite their common use, little is known about the molecular mechanisms of nZnO exposure on damaged skin. We studied the effects of topically applied nZnO particles on allergic skin inflammation in an oxazolone (OXA)-induced contact hypersensitivity (CHS) mouse model. We investigated whether exposure to nZnO during the sensitization or challenge phase would induce immunological changes and modulate transcriptional responses. We followed skin thickness, cellular infiltration, and changes in the local transcriptome up to 28 days after the challenge. The responses peaked at 24 h and were fully resolved by 28 days. Co-exposure to nZnO and hapten did not interfere with the formation of the sensitization process. Conversely, during the hapten challenge, the application of nZnO fully suppressed the development of the CHS response by the inhibition of pro-inflammatory pathways, secretion of pro-inflammatory cytokines, and proliferation of immune cells. In differentiated and stimulated THP-1 cells and the CHS mouse model, we found that nZnO particles and Zn ions contributed to anti-inflammatory responses. The immunosuppressive properties of nZnO in inflamed skin are mediated by impaired IL-1R-, CXCR2-, and LTB4-mediated pathways. nZnO-induced dermal immunosuppression may be beneficial for individuals with contact allergies who use nZnO-containing cosmetic products. Our findings also provide a deeper understanding of the mechanisms of nZnO, which could be considered when developing nanoparticle-containing skin products.

## Introduction

Use of engineered nanomaterials has been highly expanded in recent decades, leading to increased manufacturing of innovative products. Metal oxides are the most widely used types of nanoparticles that have been found in diverse commercial products.^1^ ZnO has long been used as a soothing and antiitch agent, and especially nanosized ZnO (nZnO) has been used as an active ingredient in creams and sunscreens in the personal care industry. Their excellent light scattering properties provide shimmering and effective protection against sunburn caused by UV radiation. NZnO are of greater commercial interest compared to their bulk-sized substitutes since the smaller particle size creates a more transparent and aesthetic appearance on the skin, satisfying more customer’s needs.^2^ Despite frequent dermal exposure to ZnO-containing products, little is known about the molecular mechanisms associated with topically applied nZnO in the context of allergic skin inflammation.

Contact hypersensitivity (CHS) is a delayed-type, T cell-mediated hypersensitivity response, clinically termed allergic contact dermatitis (ACD). It is estimated that CHS affects 15–20% of the global population.^3^ Common signs and symptoms of ACD include varying degrees of skin redness, itching, swelling, and blister at the site of inflammation.^4^ The CHS immune response is divided into two phases: the sensitization phase and the elicitation or challenge phase.^5^ During the sensitization phase, a low-molecular-weight molecule termed hapten acts as a sensitizer that penetrates skin and becomes immunogenic once it is covalently bound to a carrier molecule such as skin protein. This immunogenic molecule is then recognized and taken up by the skin’s antigen-presenting cells (APCs) such as epidermal Langerhans cells and dermal dendritic cells (DCs).^4^ Once APCs become activated and migrate to the nearby secondary lymphoid organ, they present parts of the processed antigen in the context of MHC class II molecules to naïve T cells residing in the lymph node.^6^ This leads to the expansion of both specific effector T cells and memory T cells. The elicitation phase is triggered upon a second skin exposure to the same hapten. This time, the educated adaptive immunity responds more quickly, resulting in T cell-mediated production of pro-inflammatory cytokines and recruitment of other frontline inflammatory cells (e.g., macrophages and neutrophils) to the site of exposure.^6^

Skin penetration by nanoparticles is an unlikely event under normal physiologic conditions, although studies have demonstrated that such penetration could occur in barrier-impaired or allergic skin.^7,8^ To date, there is a paucity of in vivo assessment of health implications from topical use of nZnO in the context of underlying ACD. Considering the high prevalence of ACD worldwide and the frequent use of nZnO-containing creams and sunscreen lotions, it is imperative to understand the interactions of nZnO with inflamed skin and the underlying mechanisms in order to address concerns related to public and occupational health. It is also not clear whether the pre-exposure to nZnO affects the development of hapten-specific immune responses. To address these issues, we studied the biological effects caused by topically applied nZnO in the context of oxazolone (OXA)-induced CHS response in mouse’s skin and investigated their mechanisms in stimulated THP-1 cells and the CHS model.

## Results

### nZnO Treatment at the Elicitation Phase Blocks OXA-Induced Ear Swelling and Skin Inflammation in a Murine Model of Contact Hypersensitivity

**Figure 1:**
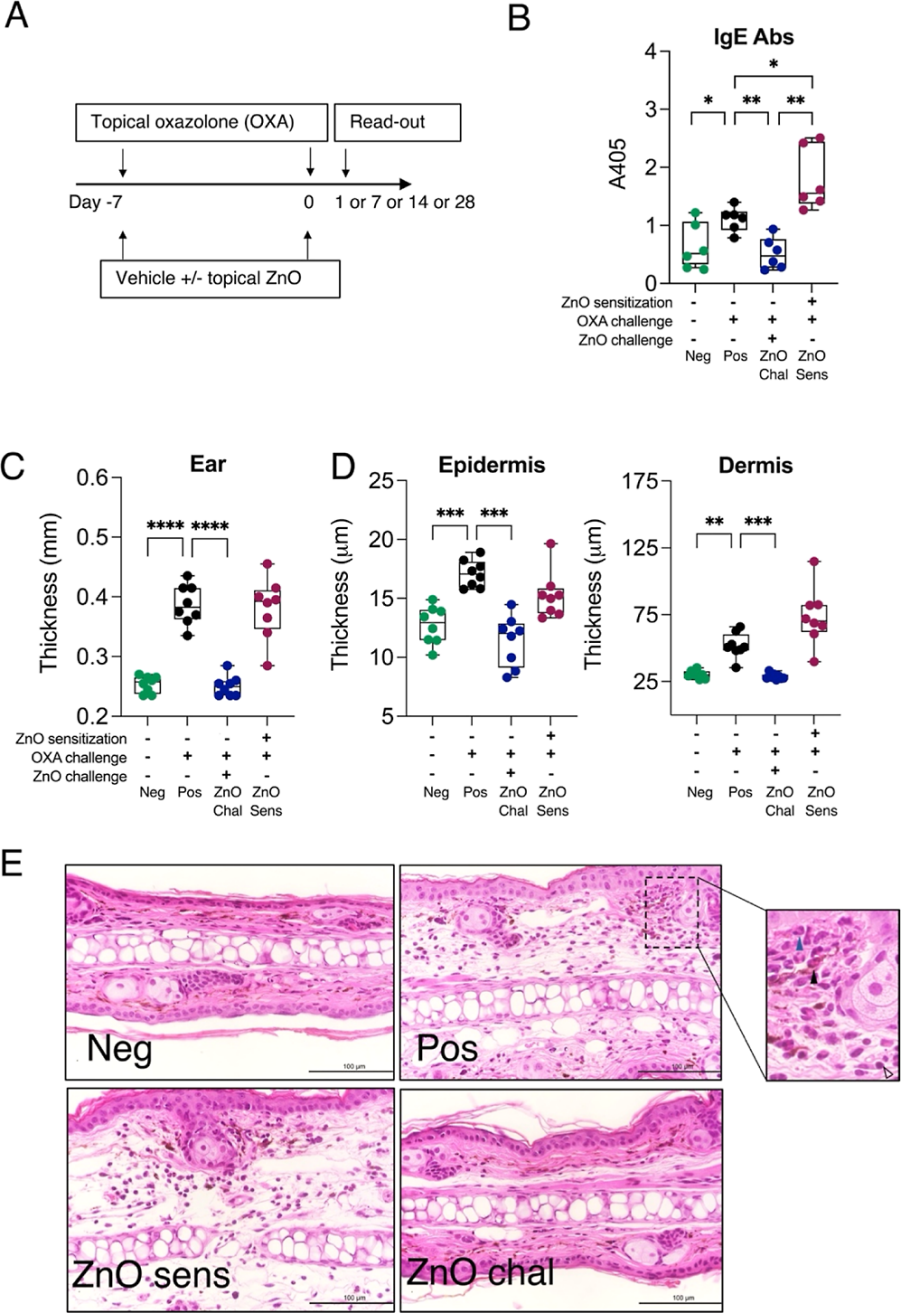
Effects of nZnO on skin thickness during the sensitization or challenge phase of CHS. (A) Mice were sensitized (day −7) and challenged (day 0) with topical application of oxazolone (OXA) with or without nanosized ZnO. (B) IgE antibody level in mice sera at day 7 after the challenge was measured by ELISA. Differences between the groups were studied by Welch’s *t*-test. (C) At 24 h after the challenge, ear thickness of OXA-sensitized mice was measured by a micrometer. (D) Thicknesses of epidermis and dermis were measured from the hematoxylin and eosin (H&E)-stained ear sections. (E) Representative images of H&E-stained epidermis and dermis of negative control (Neg), positive control (Pos), nZnO-challenged (ZnO chal), and from nZnO-sensitized (ZnO sens) mice. Blue, black, and clear arrowheads indicate neutrophil, eosinophil, and lymphocyte, respectively. Scale bar is 100 μm and used magnification 400×. Differences between the groups were studied by Brown–Forsythe and Welch ANOVA test with Dunnett’s T3 multiple comparison correction. The bars represent minimal to maximum values showing all the points. **P < 0.01; ***P < 0.001; and ****P < 0.0001.

To study the role of nZnO in the contact hypersensitivity to OXA, we sensitized mice with OXA and challenged them 1 week later with OXA with or without nZnO on dorsal sides of both ears (Figure 1A). We sought to investigate both systemic and local immune responses induced by nZnO. ELISA assays were conducted to measure the total IgG2a and IgE antibodies from the mouse sera. Significant differences were found in IgE antibody concentration between Pos vs Neg; ZnO-Sens; and ZnO-Chal, respectively, as well as between ZnO-Sens vs ZnO-Chal on day 7 (Figure 1B). On day 14 after the challenge (Figure S1A), the Pos control group still showed a higher IgE antibody level than Neg and ZnO-Chal groups. Although the IgE antibody amount in ZnO-Sens mice was decreased, it was still significantly higher than that in ZnO-Chal mice. On the other hand, no statistical differences were found in IgG2a antibody concentrations between the groups over 28 days of follow-up, although its level in OXA sensitized groups (Neg, Pos, and ZnO-Sens) seemed to increase on day 28 compared to the beginning (Figure S1A).

In the OXA-sensitized skin of mice (negative control, Neg), the vehicle treatment (acetone and olive oil) alone caused neither any changes in direct nor histological measurement of skin thickness 24 h after the challenge (Figure 1C). However, significant ear swelling and significantly thickened skin was found in the OXA-sensitized and OXA-challenged group of mice (positive control, Pos, Figure 1C). In the OXA-challenged skin sites, cotreatment with nZnO significantly reduced ear thickness as compared to the OXA alone-treated positive control group (Figure 1C). Both the overall skin thickness and dermis thickness were significantly decreased when compared to the positive control. However, this reduction did not happen when nZnO was coadministered with OXA at the sensitization phase (ZnO-Sens, Figure 1C,D). No difference was found between the ear and skin thickness measurements of OXA-nZnO cosensitized mice when compared to the positive controls (Figure 1C,D). The histological H&E staining confirmed these results (Figure 1E). The ear sections from the OXA-sensitized (Neg) and the OXA-nZnO co-challenged (ZnO-Chal) mice looked comparable with naïve mouse (naïve group data not shown), whereas the mice with the OXA-nZnO cosensitization (ZnO-Sens) showed swollen dermis resembling OXA-sensitized and challenged (Pos) mice (Figure 1E).

**Figure 2:**
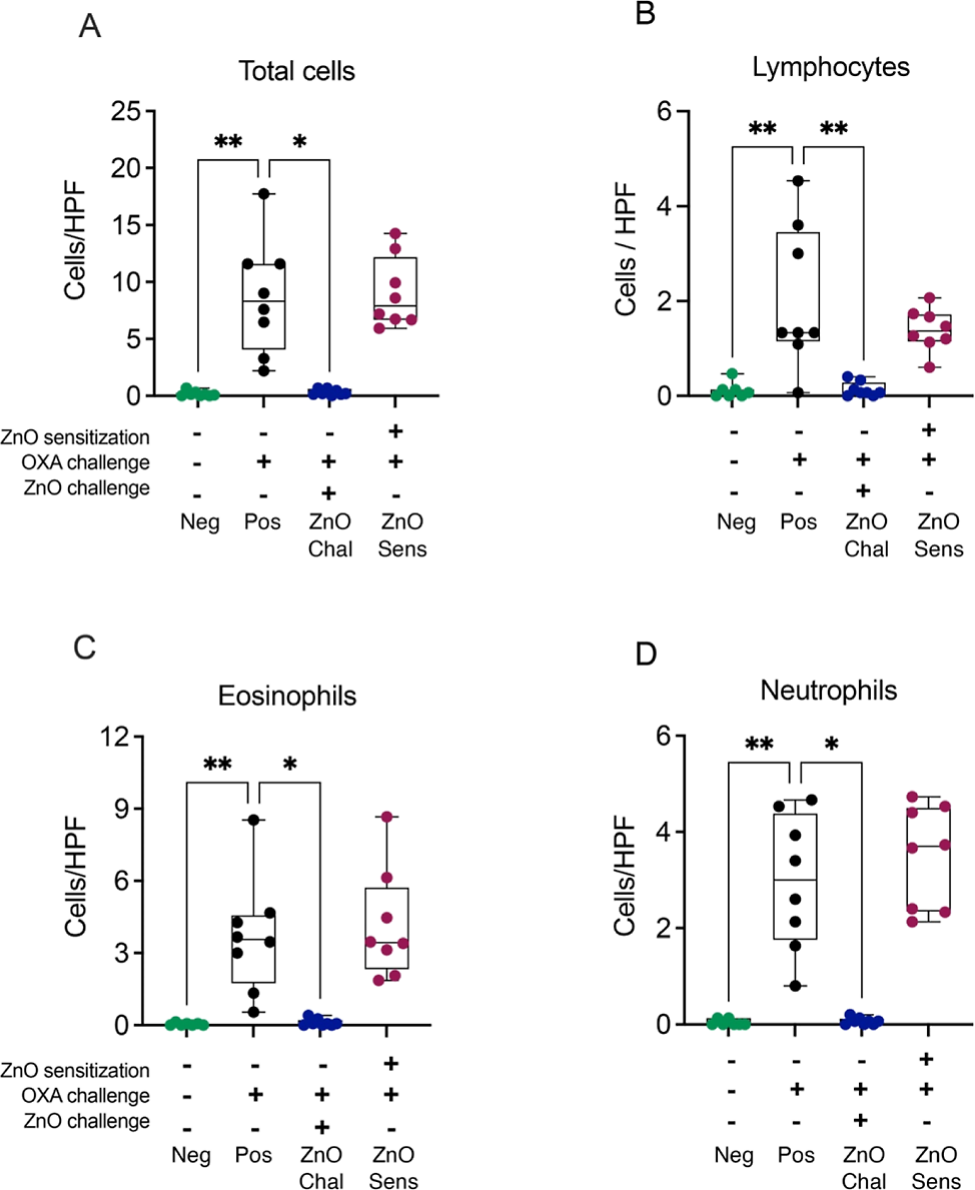
Effects of nZnO on cell infiltration at 24 h after the challenge. The number of (A) total cells, (B) lymphocytes, (C) eosinophils and (D) neutrophils were counted from the H&E-stained ear tissue sections under a light microscope at 1000× magnification high power field. Differences between the groups were studied by the Kruskal–Wallis test followed by Dunn’s multiple comparison correction, *P < 0.05; **P < 0.01. The bars represent minimal to maximum values showing all the points.

OXA sensitization and challenge (Pos) elicited significant recruitment of inflammatory cells to the ear skin as compared to nonsensitized (Neg ctrl) skin (Figure 2A–D). Dermal exposure to nZnO at the challenge phase (ZnO-Chal) reduced the numbers of lymphocytes, eosinophils, neutrophils, and total inflammatory cells close to zero as compared to the OXA-challenged mice (Figure 2A–D). In contrast, the skin of the nZnO-sensitized group exhibited an increased number of inflammatory cells, comparable to those observed in allergic skin (Pos) (Figure 2A–D). An additional mouse group received both nZnO sensitization and nZnO challenge treatment (ZnO-Sens&Chal) and the mice did not develop CHS responses, as seen in the unchanged whole ear thickness (Figure S1B), epidermal and dermal thickness (Figure S1C), and numbers of recruited immune cells (Figure S1D), altogether mirroring the Neg control. Combined, these observations propose that nZnO suppresses elicitation but does not interfere with the sensitization process in the CHS.

We also tested whether a dose–response was observed in the anti-inflammatory effect of nZnO. We found that a 10-fold lower dose (0.68 mg per ear) of nZnO, given at the time of challenge, failed to suppress ear swelling after the OXA challenge, compared to the positive control (Figure S1B). Based on actual cell counting and deconvolution analysis (Figure S2A,B), such a lower dose did not reduce the numbers of immune cells, including eosinophils, neutrophils, T cells, monocytes, macrophages, DCs, and natural killer (NK) cells, compared to the positive control. On the other hand, mice that received the original dose of nZnO (6.8 mg) at the challenge exhibited significantly lower numbers of these cells, as evidenced in the deconvolution analysis (Figure S2B), indicating an abolished immune cell migration and recruitment process.

### Time Course of Inflammatory Response in CHS

We followed the changes of CHS responses over a 28 day period post challenge (Figure S3). At the site of exposure, ear swelling peaked on day 1 and persisted until 7 days after the OXA challenge in sensitized mice (Pos group) and nZnO-sensitized groups (ZnO-Sens) (Figure S3A). The swelling decreased over time, and by day 28, it had completely resolved (Figure S3A). Neither the nZnO-challenge group nor the unchallenged negative group (Neg group) exhibited any thickening of the whole ear or dermis (Figure S3A,B). Although whole ear thicknesses appeared comparable, nZnO during sensitization (ZnO-Sens) significantly enhanced dermis thickening compared to the OXA treatment alone (Pos group) (Figure S3B). Furthermore, this thickening resolved more slowly in the ZnO-Sens group than in the positive control mice (Figure S3B).

We also saw statistically significant increases in numbers of inflammatory cells infiltrated into the dermis layer in Pos and ZnO-Sens groups at day 1 (Figure S4A–D). The significant differences in numbers of neutrophils and eosinophils were also perceived on day 7 (Figure S4B,C).

### nZnO Challenge Abrogates OXA-Induced Gene Expression in the Mouse Ear

To identify the nZnO-driven transcriptional changes, we studied genome-wide gene expression from mouse ear skin biopsies by NGS at four different time points (days 1, 7, 14, and 28) after the OXA challenge (Table 1). OXA sensitization and challenge elicited strong expression of differentially expressed genes (DEGs) to the mouse ear skin compared to the negative control mice. At day 1 and day 7, there were 4315 and 873 DEGs, respectively, in the Pos vs Neg contrast. On day 14 and day 28, much fewer DEGs, 270 and 19, were found, respectively. The number of DEGs between positive control and ZnO-Chal was also the greatest on day 1 (4496), followed by day 7 (498), with very few DEGs induced on day 14 (8), and day 28 (1). Lastly, when we compared the gene expression pattern between mice cosensitized with ZnO and OXA (ZnO-Sen) and mice sensitized with OXA alone (Pos), hardly any genes were induced. Only 21 DEGs appeared 1 day after the elicitation of CHS, while 0 or 1 ZnO-Sen-specific DEG showed up for the rest of the time points.

**Table 1:**
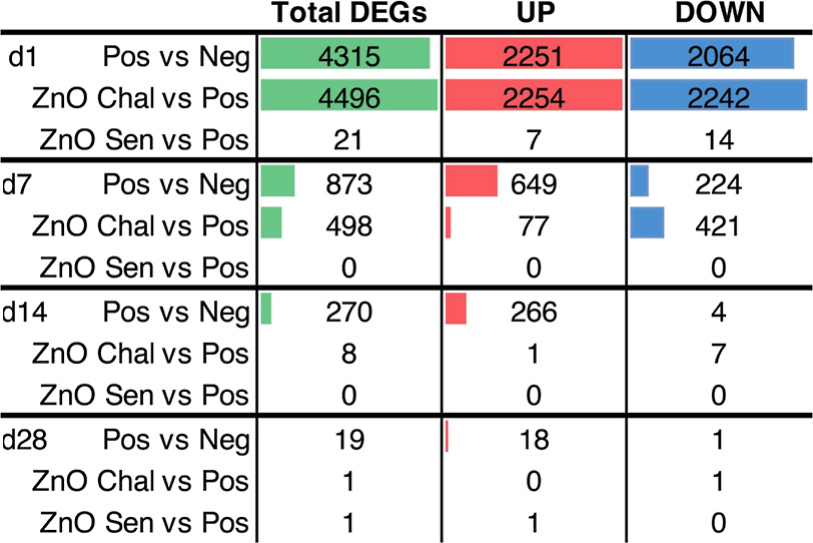
Number of DEGs at 1, 7, 14, or 28 days after the hapten challenge

### Pathway Analyses on Day 1 and Day 7 after the Challenge

**Figure 3:**
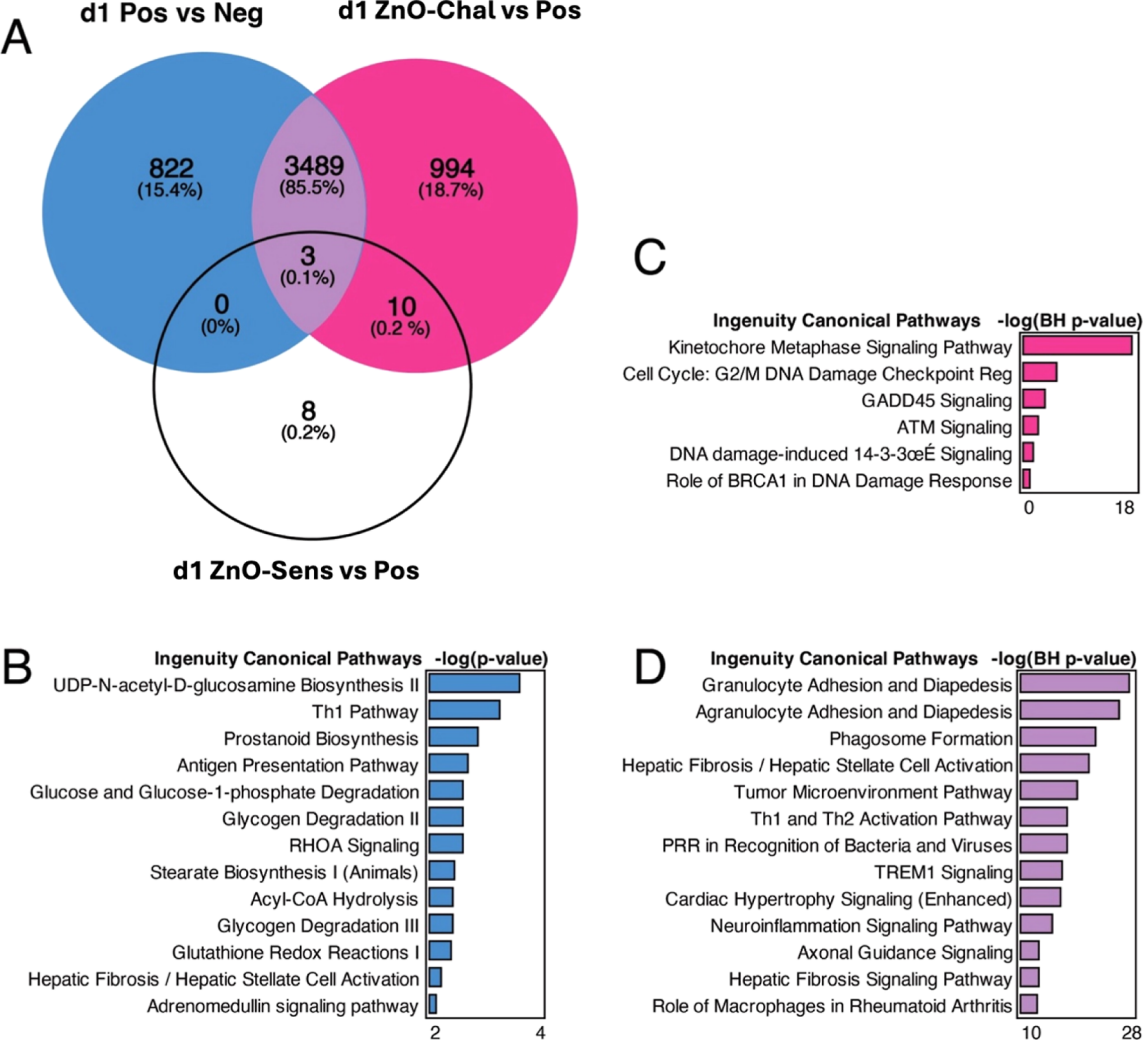
VENN-diagram of DEGs and associated pathway analyses of comparison-specific or shared DEGs at day 1. (A) VENN diagram shows that most of the DEGs (85.5%) are shared between Pos vs Neg and ZnO-Chal vs Pos comparisons. The ingenuity canonical pathways were studied on (B) 822 Pos vs Neg -specific DEGs, (C) 994 ZnO-Chal vs Pos -specific DEGs, and (D) 3492 shared DEGs within all groups. The negative logarithm of the *P*-value, obtained either from the Benjamini–Hochberg correction for multiple testing or from Fisher’s exact test, is shown for each enriched pathway.

To study the treatment-specific DEGs, we did a VENN analysis and examined the associated pathway enrichment of the specific or shared genes at day 1 (Figure 3). OXA challenge led to 822 specific DEGs (Pos vs Neg), which were enriched to helper T cell differentiation (especially to Th1), antigen presentation, and biosynthesis/degradation of glucose derivatives (Figure 3A,B) in the ingenuity pathway analysis (IPA). NZnO exposure during the challenge induced 994 specific DEGs (ZnO-Chal vs Pos), which played a role in metaphase signaling and DNA damage checkpoint regulation, GADD45 signaling, and ATM signaling (Figure 3A,C). The shared DEGs between the comparisons of Neg vs Pos and ZnO-Chal vs Pos dominated the total population of DEGs found (3489 DEGs), accounting for 85.5% of all genes. These common DEGs were associated with granulocyte adhesion and diapedesis, PRR in recognition of bacteria and viruses, Th1 and Th2 activation, and TREM1 signaling (Figure 3A,D). In addition, there were 8 genes that were exclusive to the comparison between ZnO-Sens and Pos, and 10 genes shared with comparison between ZnO-Chal and Pos (Figure 3A) but these DEGs were not enriched to any ingenuity pathway. Only 3 DEGs (Gucy1a1, Colec11, and Nrep) were common to all three comparisons (Figure 3A).

On day 7, Pos vs Neg had 486 specific DEGs, ZnO-Chal vs Pos had 111 specific genes, and like day 1, a large number of DEGs (39.3%) was shared between these two comparisons (Figure S5A). Pos vs Neg comparison-specific DEGs were involved in the activation of Th1/Th2 pathways and NK cell signaling (Figure S5B). Specific DEGs in the ZnO-Chal versus Pos contrast-mediated metaphase signaling pathways and Gαs signaling (Figure S5C). The shared 387 DEGs between Neg vs Pos and ZnO-Chal vs Pos were mainly enriched with granulocyte adhesion and diapedesis, crosstalk between DCs and NK cells, and Th1/Th2 activation (Figure S5D).

**Figure 4:**
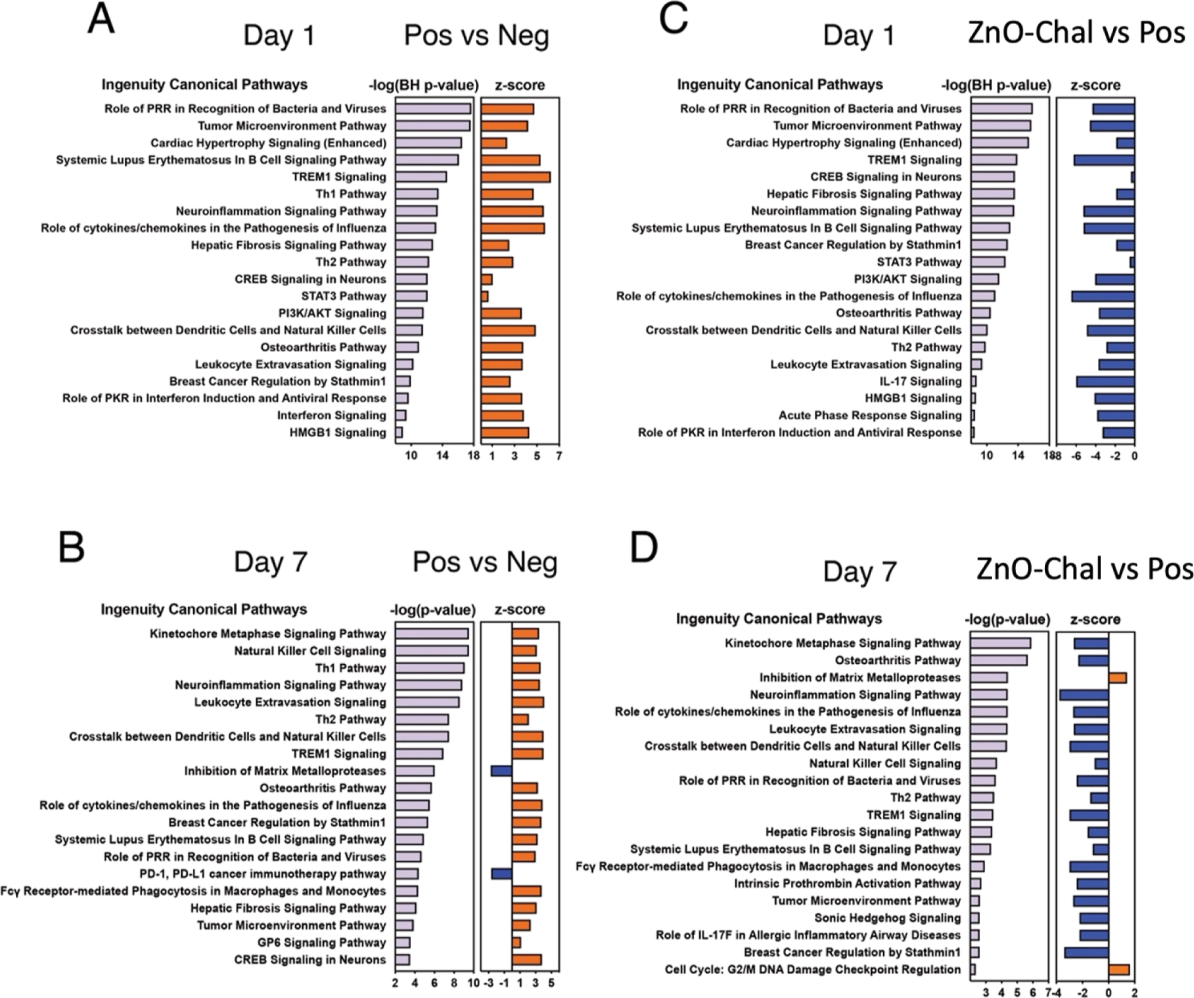
Comparison of pathway analyses of Pos vs Neg and nZnO challenge at days 1 and 7. The ingenuity canonical pathways were studied for the comparison of Pos vs Neg (A) at day 1 and (B) at day 7. The corresponding analysis was done for the comparisons between nZnO challenge vs Pos (C) at 1 day and (D) 7 days after the challenge. The negative logarithm of the *P*-value, obtained either from the Benjamini–Hochberg correction for multiple testing or from Fisher’s exact test, is shown for each enriched pathway. The *Z*-score is used to indicate whether the significantly enriched canonical pathway is activated (*Z*-score > 0) or inhibited (*Z*-score < 0).

When Pos vs Neg (Figure 4A,B) and ZnO-Chal vs Pos (Figure 4C,D) were analyzed more in detail at days 1 and 7, several common pathways were significantly enriched in both comparisons, including role in PRR in recognition of bacteria and viruses, TREM1 signaling, Th1/2 pathway, and crosstalk between DCs and NK cells. The *Z*-scores show that the directions (up or down) of the gene regulation pathways are nearly exactly the opposite between the comparisons of Neg vs Pos (Figure 4A,B) and ZnO-Chal vs Pos (Figure 4C,D) at days 1 and 7.

**Figure 5:**
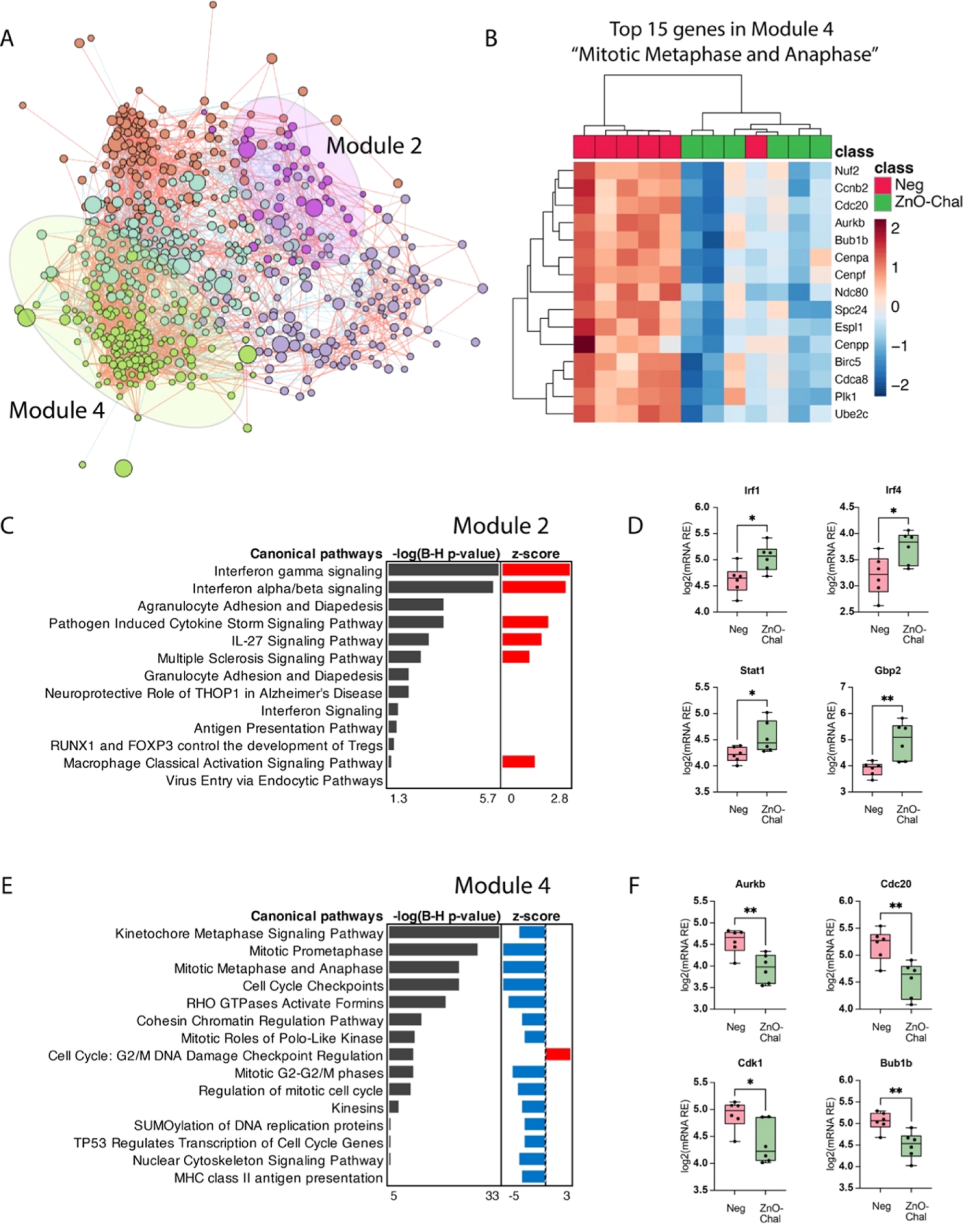
nZnO during the challenge suppresses cell division and enhances IFNγ signaling when compared to unchallenged control mice in the contact allergy model. (A) Interaction network analysis (INfORM) of ZnO-Chal vs Neg contrast yields modules 2 and 4. (B) Heatmap of ear skin gene expression of top 15 genes in module 4 by Euclidean clustering of ZnO-Chal and Neg ctrl samples. (C,E) Enrichment of canonical pathways identified in modules 2 and 4, respectively, using IPA. (D,F) Log2-transformed expression of the selected top genes in modules 2 and 4, respectively. Negative log of *P*-value from Benjamini–Hochberg correction for multiple testing is shown for each enriched pathway. *Z*-score is used to indicate whether the significantly enriched canonical pathway is activated (*Z*-score > 0) or inhibited (*Z*-score < 0). Differences between the groups were studied by unpaired *t*-test, *P < 0.05; **P < 0.01.

To investigate the possible mechanism underlying immunosuppression induced by nZnO, we performed a detailed coexpression network analysis of ZnO-Chal vs Neg DEGs on day 1. We identified five distinct network modules with modules 2 and 4 highlighted (Figure 5A). Interestingly, the top 15 genes found in module 4 were associated with mitotic metaphase and anaphase, and they showed lower expression in the ZnO-Chal group than in the negative control (Figure 5B). Genes composing module 2 enriched activated interferon gamma and interferon alpha/beta signaling pathways (Figure 5C), specifically supported by increases in Irf1, Irf4, Stat1, and Gbp2 gene expression in ZnO-Chal mice (Figure 5D). Genes found in module 4 were found to inhibit pathways such as the kinetochore metaphase signaling pathway, mitotic prometaphase, mitotic metaphase, anaphase, and regulation of mitotic cell cycle (Figure 5E), along with downregulated gene expression of Aurkb, Cdc20, Cdk1, and Bub1b in ZnO-Chal mice (Figure 5F). We additionally extracted genes associated with cytotoxicity-related pathways from the transcriptome data in module 4 of the ZnO-Chal versus Neg comparison and found that cell survivability pathways, including cell cycle genes Foxm1 and Espl1, were downregulated in the ZnO-Chal group (Figure S6).

### Immune-Suppression of nZnO and Zn Ions in LPS-Stimulated THP-1 Cells

**Figure 6:**
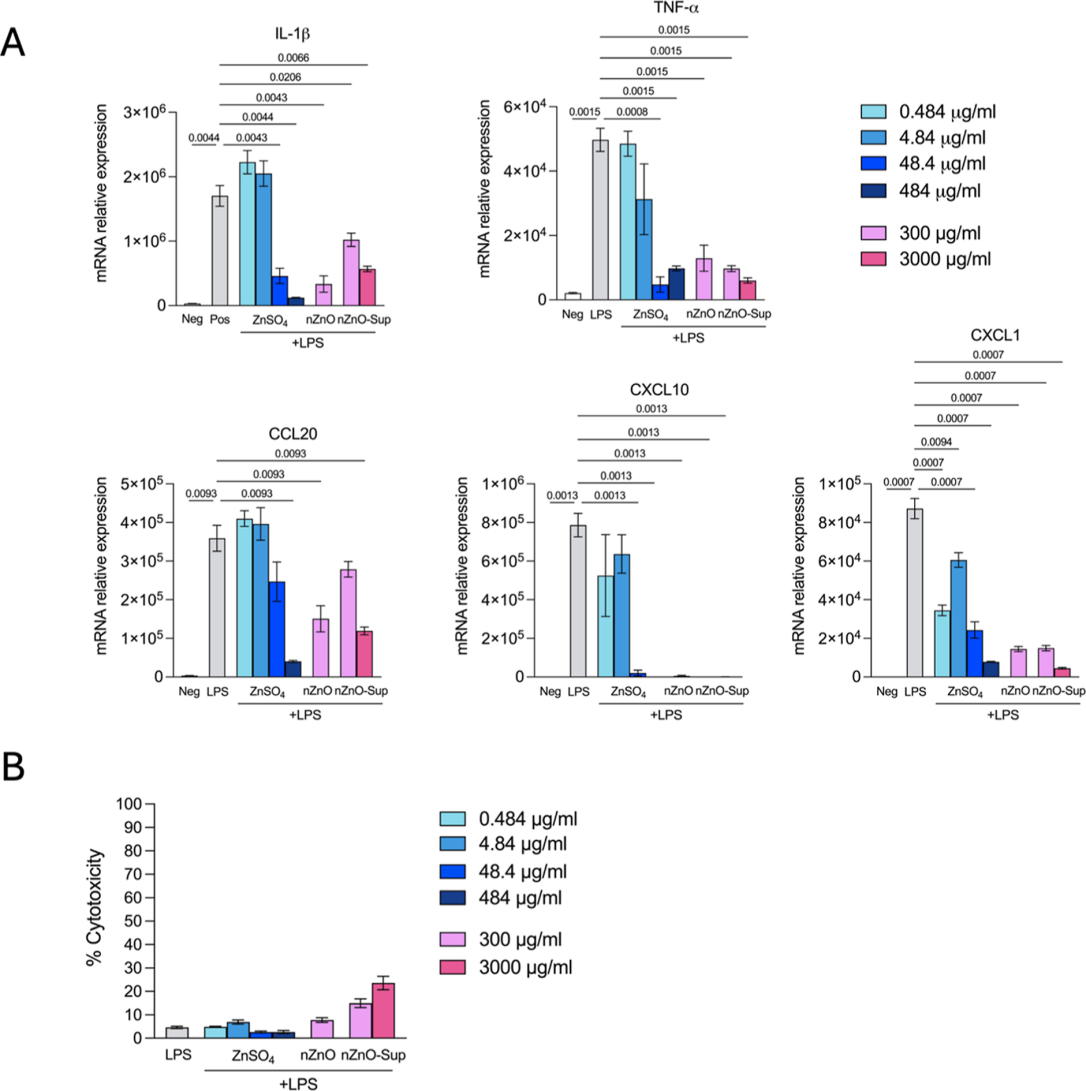
Immunosuppressive effect of nZnO and Zn ions in activated THP-1 cells. (A) Measurement of mRNA relative expression level of cytokines in PMA-differentiated THP-1 cells that were untreated (Neg), treated with LPS alone (50 ng/mL; Pos), cotreated with LPS and ZnSO_4_ (0.484, 4.84, 48.4, or 484 μg/mL), nZnO (300 μg/mL), or supernatant of nZnO (300 or 3000 μg/mL; released ionic fractions) for 7 h. (B) Cytotoxicity of differentially treated THP-1 cells quantified by lactate dehydrogenase (LDH) content released in cell supernatant. Differences among groups (*N* = 4 replicates/group) were assessed via Brown–Forsythe and Welch ANOVA tests, with multiple test correction using the Benjamini–Hochberg procedure for false discovery rate control. Statistical significance, represented as *q*-values, is displayed for *q*-values below 0.05.

Considering that ions released from nanoparticles are thought to induce cellular responses after nanoparticle exposure,^9,10^ we aimed to investigate whether this mechanism held true under inflammatory conditions. Macrophages exist in large numbers in both human and mouse skin, and macrophage-derived chemokines are important mediators for recruitment of other immune cells during skin inflammation.^9^ We first used differentiated THP-1 macrophages with LPS stimulation as a simplified in vitro model to study the effect of the Zn ions. The stimulated THP-1 cell setup is a versatile inflammation model that mimics skin-resident macrophage function while avoiding interindividual variations from the animal experiments. We found that a high dose of ZnSO_4_ (where Zn existed as Zn ions) and nZnO supernatant (contained Zn ions released from nZnO particles) significantly downregulated the expression of Il1b, Tnf, Ccl20, Cxcl1, and Cxcl10, which was similar to the suppressive effect of nZnO (contained both ZnO particles and Zn ions released) on the expression of these genes in LPS-activated THP-1 cells (Figure 6A). Subsequently, the cytotoxic capacities of different treatments were assessed, and we observed less than 10% cell death induced by ZnSO_4_ or nZnO, and around 20% cell death by the nZnO supernatant fraction (Figure 6B). These findings suggest that both nZnO particles and the Zn ions released from the particles likely cause cellular anti-inflammatory responses to LPS-stimulated differentiated THP-1 cells, largely independent of their cytotoxicity.

### nZnO May Impair IL-1R-, CXCR2-, and LTB4-Mediated Pathways in CHS Responses

Our in vitro data indicate that Zn ions are capable of suppressing inflammatory responses in cells. This prompted us to examine whether Zn ions could exert similar effects in the context of CHS inflammation in vivo. Additionally, gene analysis indicates that the expression levels of Il1r, Cxcr2, and their associated ligands and molecules (e.g., Il1b and Cxcl2/3/5) greatly differed between ZnO-Chal and Pos control groups (Figure S7A). Our heatmap and clustering analysis further show that Il1, chemokine-, and leukotriene-related genes formed distinct clusters in their expression profiles among different groups and were consistently suppressed in the ZnO challenge group (Figure S7B–D). Building on our findings and existing research regarding Zn’s inhibitory effects on LTB4 production,^11^ we aimed to investigate and compare the impacts of nZnO, Zn ions, and the impact of blocking IL-1R, CXCR2, and LTB4 in a CHS mouse model.

**Figure 7:**
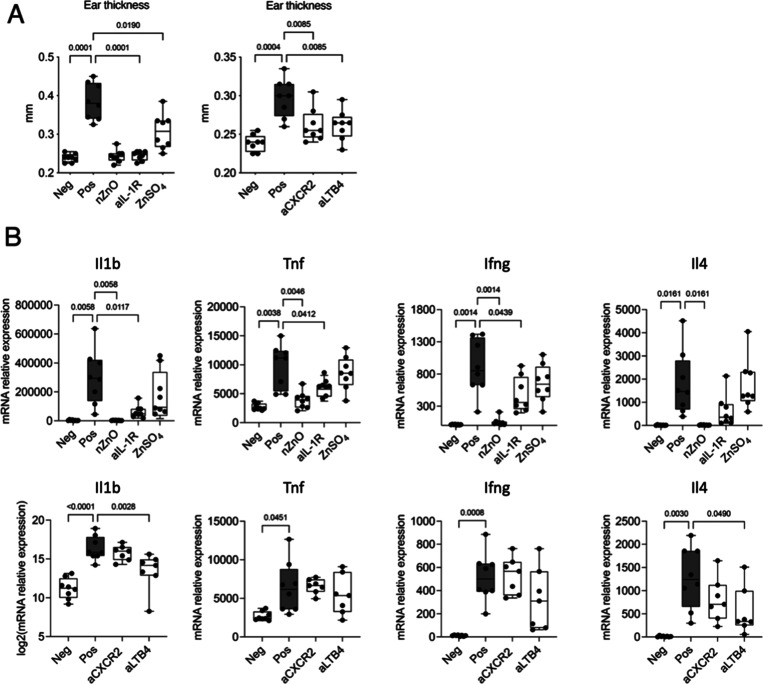
Ear thickness and target cytokines expression of nZnO-, Zn ions, or blockade-induced anti-inflammatory response in contact allergy mice. (A) Ear thickness was measured by a micrometer in nonallergic mice (Neg); allergic mice intraperitoneally treated with vehicles (Pos), nZnO, ZnSO_4_, IL-1R antagonist anakinra (aIL-1R), CXCR2 antagonist (aCXCR2), and LTB4 synthesis inhibitor (aLBT4). (B) Expression of key inflammatory cytokines in the ear was studied by qPCR. Differences among groups (*N* = 8 mice/group) were assessed via Brown–Forsythe and Welch ANOVA tests, with multiple test correction using the Benjamini–Hochberg procedure for false discovery rate control. Statistical significance, represented as *q*-values, is displayed for *q*-values below 0.05.

We observed that both nZnO and ZnSO_4_ reduced ear swelling in mice with contact allergy (Figure 7A). Mice with pharmacologically blocked IL-1R, CXCR2, and LTB4 also failed to develop CHS responses, as evidenced by their decreased ear thickness (Figure 7A). Furthermore, the expression of Il1b, Tnf, and Ifng significantly decreased when IL-1R was blocked, while Il4 expression showed borderline decreases (Figure 7B). Additionally, the expression of Il1b and Il4 decreased after blocking LTB4 compared to their respective positive controls (Figure 7B). These findings demonstrate that inhibiting IL-1R, CXCR2 signaling, and LTB4 production suppresses allergic skin inflammation in CHS. Since the effects of blocking and nZnO were similar, these data suggest that the mechanism of nZnO-induced immune suppression could be at least partially due to the inhibition of IL-1R-, CXCR2-, and/or LTB4-mediated pathways in CHS development.

## Discussion

Epidemiological meta-analyses have shown that the prevalence of contact allergy is increasing, being already even over 20% in the general, unselected population.^12^ ZnO nanoparticles are nowadays commonly incorporated into products like sunscreen cream, ointment, pigments, and therapeutic agents.^13^ Traditional ZnO cream products relieve skin rashes and itches and provide a barrier from irritating substances.^14^ The size distribution data of ZnO in commercial products are rarely studied or reported, possibly due to proprietary information. Nonetheless, our previous study has shown that different particle sizes of ZnO influence the cellular transcriptomic responses.^10^ Furthermore, incorporation of nanoparticles in skin products is increasing as it enhances the protective and aesthetic properties of the original products.^15^ Previous studies suggested that nZnO does not penetrate into the healthy skin layers in significant amounts.^8,16^ A weekly topical application of nZnO-containing sunscreens for 36 weeks was reported not to cause any histological changes in the skin or transcriptomic changes in the liver.^17^ However, in real-life, nZnO-containing creams are often spread on damaged skin areas (e.g., prolonged exposure to direct UV) or to skin, which has impaired barrier integrity due to an (allergic) inflammation.^18^

Since the local effect of topical application of nZnO in inflamed skin remains uncharacterized, we sought to provide insights into the potential effect posed by nZnO in the mouse model of CHS, which imitates one of the most common skin inflammatory conditions in humans.

ACD is characterized with an increased skin thickness due to the influx of inflammatory cells into the skin’s viable layer after repeated exposure to a specific hapten. The CHS reaction usually peaks within 24 or 48 h after re-exposure to the sensitizing hapten.^5^ In our study, we also demonstrate that the oxazolone-induced CHS responses were at peak at 24 h after the allergen challenge, drawing from changes on ear thickness, immune cell numbers, and transcriptomic profiles, with weakened reactions lasting for a week, suggesting an effective resolution program.

Interestingly, nZnO treatment given at the time of challenge fully suppressed the skin inflammation and allergen induced gene expression in the CHS model. Similar effects of nZnO were seen previously in a mouse model of atopic dermatitis,^8^ despite differences in pathogenesis (e.g., repeated ovalbumin sensitizations vs single OXA sensitization; allergen induced vs hapten induced reaction), suggesting that topically applied nZnO may exert broad anti-inflammatory responses on the skin. Consistent with this finding, our pathway analyses indicated that nZnO suppressed the presentation of the antigen-hapten complex, cell chemotaxis, and cytokine expression. The exact recognition mechanisms of haptenated antigens are poorly understood, but TLR2/4 are suggested to play a critical role in the initiation of CHS.^19^ Also, in our study, “PRR in recognition of bacteria and viruses”, “TREM1 signaling”, and also “crosstalk with DCs and NK cells” were listed in the top-ranked canonical pathways and highly downregulated (with negative *z*-score) after nZnO-treatment during challenge in the pathway analyses, supporting that topical nZnO given at the time of allergen challenge fully suppresses elicitation of contact allergic inflammation. Supporting the in vivo finding, nZnO was able to inhibit pro-inflammatory cytokine gene expression in LPS-stimulated THP-1 cells. The dose we used is higher than those reported in the literature,^20^ but we aimed to mimic the concentration used in vivo. Additionally, our deconvolution analysis revealed reduced populations of innate and adaptive immune cells upon nZnO exposure during the challenge phase. In our model, the nZnO-Chal group received the sensitizing agent oxazolone at the same time during the challenge phase (no inflammation elicited yet), supporting the idea that inflammatory cell recruitment is somehow stopped. Our Figure 2 shows the actual cell counts of lymphocytes, neutrophils, and eosinophils, indicating a lack of infiltrating leukocytes in the ZnO-Chal group. These suggest a paralyzed state of effector immune cell recruitment caused by nZnO, subsequently leading to suppressed inflammatory mediator release.

DEGs specific for the nZnO-Chal group were mostly enriched to cell division (Kinetochore Metaphase Signaling) and in lesser amount also to DNA-damage checkpoint regulation through GADD45 and ATM signaling. Growth arrest and DNA damage-inducible 45 (GADD45) is reported to maintain the genomic stability via modulating the G2/M cell cycle checkpoint,^21^ and ATM (ataxia telangiectasia mutated) is known to trigger the activation of DNA damage checkpoint and the subsequent cell cycle arrest and DNA repair.^22^ Our network analyses of DEGs found between nZnO-Chal vs Neg also give hints to the underlying immunosuppressive mechanism of nZnO. The significant activation of the IFN signaling pathway and its related upregulated genes indicates a potential role of nZnO in antiviral and immune defense mechanisms, which aligns with the previous finding of nZnO on differentiated THP-1 cells.^10^ The exceptionally strong inhibition of pathways related to mitosis and cell cycle progression suggests that nZnO exposure significantly disrupts normal cell division processes. This disruption could lead to a decrease in cell proliferation within the exposed tissue. Furthermore, the activation of the G2/M DNA Damage Checkpoint Regulation pathway suggests that cells might be halting their progression through the cell cycle at the G2/M checkpoint in response to nZnO exposure. Some medications, such as azathioprine for rheumatoid arthritis and ulcerative colitis, exert an immunosuppressive effect by acting as a purine analogue and an inhibitor of DNA synthesis.^23^ A somewhat similar mode of action could be related to the immunosuppressive effect of nZnO, which could be further explained by the inhibition of cell survivability pathways, which potentially lead to impaired cell cycle progression, reduced capacity of DNA repair, and metabolic dysfunction, thus increasing cell death.

In sharp contrast, nZnO given during the sensitization phase had no effect on allergen-induced skin inflammation, suggesting that exposure to nZnO during the first encounter with the hapten has no effects on the sensitization process itself including protein haptenization or activation of specific T cells. Interestingly, the cosensitization with nZnO almost doubled the thickening of the dermis 1 day after the challenge, and it also took longer to resolve the ear swelling. Based on our cell counting, this was not due to the enhanced cellular infiltration of lymphocytes, eosinophils, or neutrophils. Therefore, it might be that cosensitization with nZnO induces nonspecific innate immunity responses initiated by the skin keratinocytes and leading to temporary thickening of the dermis after allergen challenge. The excess of reactive oxygen species in the skin has been shown to induce an augmented breakdown of hyaluronic acid (HA).^24^ The formed HA fragments are able to activate DCs via toll-like receptor (TLR) 2- and TLR4-mediated molecule recognition,^25^ thereby enhancing a nonspecific skin inflammation. Additionally, while Ilves et al. reported an elevated level of total IgE when nZnO was applied onto mouse atopic dermatitis skin,^8^ we saw an increase in total IgE concentration when nZnO was given at the sensitization phase in the CHS model compared to the positive control, but a decrease in its level when nZnO was given at challenge. Although a mechanistic understanding is much needed, our findings point to a possibility that released Zn ions from nZnO or the particles themselves could differentially affect the IgE production capacity of B cells, depending on the immune context. Further insights into the translocation of nZnO and/or Zn ions into the skin are needed, and these dynamics could be critically dependent on factors such as the time of exposure, length of exposure, particle size, and release rate of Zn^2+^.

Metal nanoparticles are able to release metal ions that could bind to the membrane or intracellular proteins or lipids and subsequently cause protein dysfunction, excessive oxidative stress, and mitochondrial defect.^26^ Upon nanoparticles entering the cells, their biological behaviors are associated with their ion dissolution property,^27^ which was also corroborated by our in vitro findings. In the context of LPS-induced acute inflammation, we demonstrated that dose-dependent immune suppression was associated not only with nZnO but also with the ionic form of Zn, whether initially in the aqueous state or dissolved from the nanoparticle dispersion. Consistent with this, Zn ions significantly reduced ear swelling in the mouse experiments. Our results underscore the vital mechanistic role of Zn ions in the anti-inflammatory responses elicited by nZnO.

In the presence of pharmacologic blockers to IL-1R, CXCR2, and LTB4, contact allergic reactions were inhibited significantly, mirroring the effect of nZnO given at the time of challenge in CHS. Macrophage-secreted IL-1α and CXCL2 (ligand for IL-1R and CXCR2, respectively) have been demonstrated to drive the DC cluster formation and migration, which are essential for complete elicitation of local T cell responses and inflammation in CHS skin.^28^ LTB4 eicosanoid, converted from LTA4 by the Zn-dependent enzyme LTA4H, fuels neutrophil recruitment to the skin in allergic dermatitis.^29^ Our data point to the possibility that nZnO may cause defects in the IL-1R-, CXCR2-, and LTB4-mediated signaling pathways, thereby impeding leukocyte migration and thwarting contact allergic responses.

## Conclusions

This study aimed to evaluate the effects of topical nZnO in the sensitization and elicitation of CHS response via histological, molecular, and transcriptomics approaches. Our results show that coexposure to nZnO and hapten does not interfere with the sensitization process, while during the hapten challenge, application of nZnO fully suppresses the development of the CHS response via inhibition of the pro-inflammatory pathways, secretion of pro-inflammatory cytokines, and proliferation of immune cells. The immunosuppressive properties of nZnO in sensitized and inflamed skin could be mechanistically explained by the action of particles themselves and Zn ions released impairing cell cycle processes and IL-1R-, CXCR2-, and LTB4-associated pathways. Our findings provide information for people who have developed a contact allergy (e.g., nickel allergy cases) or other inflammatory skin diseases.

## Materials and Methods

### Mice

Female C57BL/6j mice were purchased from Envigo (The Netherlands) and acclimatized for 1 week. The mice were 8 weeks old at the start of the experiment. The mice were housed in groups of four in transparent plastic cages bedded with an aspen chip and were provided with a standard mouse chow diet and tap water ad libitum when not being treated. The environment of the animal room was carefully controlled, with a 12 h dark–light cycle, a temperature of 20–21 °C, and a relative humidity of 40–45%. The experiments were performed in agreement with the European Convention for the Protection of Vertebrate Animals Used for Experimental and Other Scientific Purposes (Strasbourg March 18, 1986, adopted in Finland May 31, 1990). All animal procedures were approved (ESAVI/518/04.10.07/2017 and ESAVI/35434/2022) by the Social and Health Services of the State Provincial Office of Southern Finland.

### nZnO Particles and Suspension Preparation

ZnO nanoparticle powders were purchased from Nanostructured & Amorphous Materials Inc. (Houston, TX). Characterization of the particles has been conducted and shown in our previous study^10^ and is provided in Tables S1 and S2. NZnO suspensions used for the skin sensitization and elicitation reactions were prepared by weighing the materials into glass tubes, dispersing in oxazolone/acetone/olive oil mixture, and vortexing. Immediately before application to the backs or ears of mice, the suspensions were vortexed again. To investigate the role of Zn^2+^ (later in the text referred as Zn) ions in CHS, another group of mice received ZnSO_4_ (11 mg/treatment; Sigma-Aldrich) instead of nZnO on the ear at challenge.

We used a realistic dose of nZnO for the in vivo experiments (6.8 mg/treatment), which was selected based on the EU decision released in 2016 regarding the use of nZnO as a UV filter in cosmetic products. The EU statement states that both bulk and nano forms of ZnO should be allowed to use at a maximum concentration of 25% (w/w) in the sunscreen lotion.^30^ To study the effect of nZnO at a lower dose, an additional group of mice receiving 0.68 mg of nZnO per treatment was included.

### Animal Treatment Protocol

Mice were treated as previously described.^31^ Briefly, for the sensitization protocol, 50 μL of OXA (10 mg/mL) dissolved in vehicle (acetone/olive oil, 4:1, v/v) was pipetted onto the shaved and gently tape-stripped back skin under anesthesia. One week later, mice were anesthetized and challenged, and 25 μL of OXA (3 mg/mL) was pipetted and spread onto the dorsal side of both ears. After 24 h, 7 days, 14 days, and 28 days, the mice were killed by isoflurane overdose (Figure 1A). Ear thickness was measured with a micrometer (Mitutoyo, Kanagawa, Japan) and each ear was measured twice. Ear tissues were harvested, stabilized in RNAlater solution (Life Technologies Ltd., Paisley, UK), and stored at −80 °C for total RNA isolation and transcriptome analysis.

### Serum Antibody Measurements by ELISA

Total IgE and IgG2a levels in sera were analyzed by sandwich ELISA using a standard BD Pharmingen protocol. Briefly, plates were coated with 2 μg of antimouse IgE (BD Pharmingen 553413, clone R35–72) and antimouse IgG2a (BD Pharmingen 553446, clone R11–89) in 0.05 M NaHCO_3_ (pH 9.6) and incubated overnight at 4 °C. On the following day, the plates were washed with PBS-Tween 20 (0.05%) and blocked (3% BSA/PBS) for 1 h at room temperature. After washing, serial dilutions of sera (1:10, 1:50) in dilution buffer (1% BSA/PBS) were added and incubated overnight at 4 °C. After washing, biotin-conjugated rat antimouse IgE (BD Pharmingen, cat. 553419, clone R35–118) or IgG2a (BD Pharmingen, cat. 550332, clone R19–15) monoclonal antibody (2 μg/mL) were incubated for 1 h at room temperature. Subsequently, streptavidin-horseradish peroxidase HRP) (BD Pharmingen, cat. 554066) was diluted to 1/2000 in 1% BSA/PBS. After washing the plate six times, streptavidin-HRP was added for 30 min at room temperature. Finally, the ABTS substrate solution (Thermo Scientific, cat. 37615) was added to washed plates, and optical densities were read with an automated ELISA reader at 405 nm (Labsystems Multiskan, Thermo Electron).

### In Vivo Blocking Experiment

For blocking experiments, OXA-sensitized and -challenged mice received an intraperitoneal injection of 30 mg of IL-1R antagonist Anakinra (Kineret; Biovitrum AB, Stockholm, Sweden) at 17 h before and at the time of the OXA challenge; 50 μg of CXCR2 inhibitor (SB265610; Tocris Bioscience) or 0.4 mg of leukotriene B4 (LTB4) synthesis inhibitor Bestatin (Cayman Chemical) at 6 h before and at the time of the OXA challenge; or their corresponding vehicle controls (PBS or 5% DMSO in PBS). After 24 h, the mice were euthanized, and ear samples were collected for further analysis.

### Histology

In the histological analysis, part of the ear biopsy was fixed in 10% buffered formalin and embedded in paraffin and then 4 μm skin sections were cut and stained with hematoxylin and eosin (H&E) to measure epidermal and dermal thickness at 100× magnification. Inflammatory cells in the dermis were counted in 15 high-power fields at 1000× magnification.

### RNA Extraction RT-PCR

Ear samples were homogenized in 1 mL of TRIsure reagent (Bioline Reagents Ltd., London, UK) with an Ultra-Turrax homogenizer. The RNA extraction was performed following the instructions provided by Bioline Reagents. The samples were treated with DNase I and cleaned up by a NucleoSpin RNA Clean-up XS (Macherey-Nagel-N, Düren, Germany) to remove any possible residues of organic solvents. The concentration and integrity of isolated RNA was determined by a NanoDrop spectrophotometer (ND-1000, Thermo Fisher Scientific Inc., Wilmington, NC, USA) and Bioanalyzer 2100 (Agilent, Santa Clara, United States), respectively.

Complementary DNA (cDNA) was synthesized from 500 ng of total RNA in a 25 μL reaction using Multi-Scribe Reverse Transcriptase and random primers (The High-Capacity cDNA Archive Kit, Applied Biosystems, Foster City, CA, USA) according to the manufacturer’s protocol. The synthesis was performed in a 2720 Thermal Cycler (Applied Biosystems, Carlsbad, CA, USA) starting at 25 °C for 10 min and continuing at 37 °C for 120 min. Primers and probes for PCR analysis were ordered from Applied Biosystems. The PCR assays were performed in 96-well optical reaction plates with Relative Quantification 7500 Fast System (7500 Fast Real-Time PCR system, Applied Biosystems) by the manufacturer’s instructions. Amplifications were done in an 11 μL reaction volume containing TaqMan universal PCR master mix and primers provided by Applied Biosystems and 1 μL of cDNA sample. Ribosomal 18S was used as an endogenous control.

### RNASeq

Conversion Software (bcl2fastq2) was used to convert BCL files to the FASTQ file format and demultiplex the samples. Sequenced reads were trimmed for adaptor sequence and masked for low-complexity or low-quality sequence using Trimmomatic (parameters: LEADING:3, TRAILING:3, SLIDINGWINDOW:4:15, and MINLEN:36). Trimmed reads were mapped to the GRCm38.p6 whole genome (GENCODE *Mus musculus* Release M25) using STAR aligner (2.6.0c). Counts per gene were calculated using featureCounts software (v1.6.4). Differential expression analysis was done in the DESeq2 software in the R environment. The count values were normalized between samples using a geometric mean. Sample-wise factors were estimated to correct for library size variability and estimation of dispersion (i.e., variance and scatter) of gene-wise values between the conditions. Negative binomial linear models and Wald tests were used to produce *p*-values. Low-expression outliers were removed using Cook’s distance to optimize the *p*-value adjustment, and finally, multiple testing adjustment of *p*-values was done with the Benjamini–Hochberg procedure.

### Pathway Enrichment Analyses

To evaluate the distribution of differentially expressed genes between specified contrast sets, Venn diagrams were created using Venny.^32^ The list of DEGs from Pos/Neg controls, ZnChal/Pos, and ZnSens/Pos contrast were submitted into the IPA tool (QIAGEN Inc., Venlo, The Netherlands, https://digitalinsights.qiagen.com/IPA, version 65367011) to study the enriched canonical pathways.^33^*Z*-scoring assessment using the corresponding expression fold-change of each comparison was then used to predict whether the identified significantly enriched functions are activated or inhibited. Where applicable, we refer to a significantly enriched downstream biological process as activated if the *z*-score > 0 or inhibited if the *z*-score < 0. *Z*-score = 0 implies that the direction of the effect is unknown or ambiguous. *Z*-scores that exceed ±2 are highly predictive of an activated or inhibited biological process.

Inference of Network Response Modules (INfORM) method was applied to generate coexpression networks and identify gene modules for the ZnO Chal vs Neg contrast. Multiple gene coexpression networks were inferred from gene expression profiles using various network inference algorithms, which were then combined to form an ensemble gene network, retaining only high-confidence edges. Gene modules were identified within this network using the Walktrap community detection algorithm. The detailed methodology for gene network module detection is outlined in Marwah et al.^34^ Subsequently, enriched canonical pathways associated with these gene modules were identified using IPA. Pathway categories were selected based on a Benjamini–Hochberg adjusted *p*-value of ≤0.05.

Partial least-squares discriminant analysis was performed to visualize difference between genes related to Organismal Death in gene module 4 of the ZnO Chal vs Neg contrast.

### CIBERSORT Deconvolution of Immune Cell Subtypes

The CIBERSORT^35^ deconvolution analysis was employed to predict immune cell populations in the mouse ear bulk RNA-seq data set using the mouse orthologs of DerM22 and LM22 gene signature matrices.^36^ The estimations are based on 500 permutations, as suggested by CIBERSORT.

### Heatmap and Clustering of Genes

The terms “chemokine”, “chemokine receptor”, “IL1”, “IL1 receptor”, and “leukotriene” were used in Enrichr term search.^37^ The gene lists from pathway search results, except for the “Virus-Host PPI P-HIPSTer 2020” library, were used to determine which genes were present in the previously obtained differentially expressed gene matrixes. Using the R package p heatmap,^38^ the matching genes whose *p* < 0.05 were included for visualization. The normalized gene expression counts were clustered with the Ward D2 method using Euclidean distances. The dendrogram was clustered using the R internal “cutree” function.

### Cell Culture

The THP-1 (human leukemia monocytic cells) cell line was purchased from American Type Culture Collection (ATCC, Rockville, MD, USA). Cells were grown in an RPMI medium supplemented with 10% fetal bovine serum (FBS), 1% GlutaMAX, 1% HEPES, 0.05 mM 2 ME, and 1% PEST at 37 °C under a humidified atmosphere of 5% CO_2_. RPMI 1640, FBS, GlutaMAX supplement, 4-(2-hydroxyethyl)-1-piperazineethanesulfonic acid (HEPES), and penicillin–streptomycin (PEST) were purchased from Gibco, Life Technologies (NY, USA). 2-Mercaptoethanol (2-ME) was obtained from Sigma-Aldrich (CA, USA). Prior to treatment with nZnO or ZnSO_4_, cells were grown in a medium with 50 nM PMA for 48 h to induce macrophage differentiation. PMA-containing RPMI medium was refreshed once after the first 24 h. Cell passages used were between 7 and 10.

Differentiated THP-1 cells were then first treated for 1 h with ZnSO_4_ (0.484, 4.84, 48.4, or 484 μg/mL; Sigma-Aldrich), nZnO (300 μg/mL) suspension, or supernatant of nZnO-conditioned RPMI media. The supernatant was prepared by incubating nZnO particles (300 or 3000 μg/mL) in complete RPMI for 8 h, followed by 20,800*g* centrifugation at 4 °C for 10 min and again for 50 min to sediment nZnO particles. nZnO stock and diluted suspensions in RPMI were sonicated in an Elmasonic S15H bath sonicator at 30 °C for 20 min and briefly vortexed immediately before adding to the cells. After 1 h of pretreatment, cells were stimulated with 50 ng/mL purified LPS (*Escherichia coli* 0111: B4, Sigma-Aldrich) for additional 6 h. Untreated and only LPS-stimulated differentiated THP-1 cells were taken as negative and positive controls, respectively. After 7 h, THP-1 cells were harvested and homogenized in a lysis buffer and extracted using a RNeasy Mini kit (Qiagen, Germany) according to the manufacturer’s protocol.

### Cytotoxicity Measurement

Cytotoxicity in THP-1 cells was determined by measuring the amount of LDH released from cell membrane into the culture medium using a Cytotoxicity Detection KitPLUS (LDH) Kit (Roche, Switzerland) according to the manufacturer’s instruction. Briefly, after 7 h treatment, cell supernatant was collected and mixed with LDH detection reagent. After 30 min of incubation, the absorbance of sample was measured at 490 nm target wavelength and 620 nm reference wavelength using a microplate reader.

### Data Analysis

The differences between the groups were analyzed using the one-way or two-way ANOVA, followed by Tukey’s test or by Kruskal–Wallis test, followed by Dunn’s multiple comparison correction or by Brown–Forsythe and Welch ANOVA tests, followed by the Benjamini–Hochberg procedure for false discovery rate control or by Mann–Whitney test in Graph Pad Prism program, unless otherwise specified in the figure legend. The data are expressed as mean values ± SEM except as otherwise provided. *P < 0.05; **P < 0.01; ***P < 0.001; and ****P < 0.0001.

## Data Availability

The data that support the findings of this study are openly available from the authors after request.

## Supporting Information Available

Sera antibody measurement, dose response of nZnO in mice, effect of nZnO in mice at different time points, pathway analysis of ZnO-Chal vs Neg associated genes, and most DEGs between ZnO-Chal vs Pos (PDF)
