# Supplementary material for: ZnO Nanoparticles as Potent Inducers of Dermal Immunosuppression in Contact Hypersensitivity in Mice
Source: ACS Nano. 2024 Oct 14;18(43):29479–91. doi: 10.1021/acsnano.4c04270 (PMC11526425; doi:10.1021/acsnano.4c04270)
Supplement: Supplementary file 1 — nn4c04270_si_001.pdf [file nn4c04270_si_001.pdf]

Supporting Information for Publication

ZnO nanoparticles as potent inducers of dermal immunosuppression in contact hypersensitivity in mice

Shuyuan Wang[1,2], Marit Ilves[2§], Kuunsäde Mäenpää[2§], Lan Zhao[2], Hani El-Nezami[1,3], Piia Karisola[2*], Harri Alenius[2,4*]

[1] School of Biological Sciences, University of Hong Kong, Pok Fu Lam Road, Hong Kong
[2] Human Microbiome Research Program, University of Helsinki, Haartmaninkatu 3, 00290 Helsinki, Finland
[3] School of Medicine, Institute of Public Health and Clinical Nutrition, University of Eastern Finland, P.O. Box 1627, 70211 Kuopio, Finland
[4] Institute of Environmental Medicine (IMM), Karolinska Institutet, Stockholm 171 77, Sweden

[§]The authors share the second position.
[*]Address correspondence to piia.karisola@helsinki.fi and harri.alenius@helsinki.fi

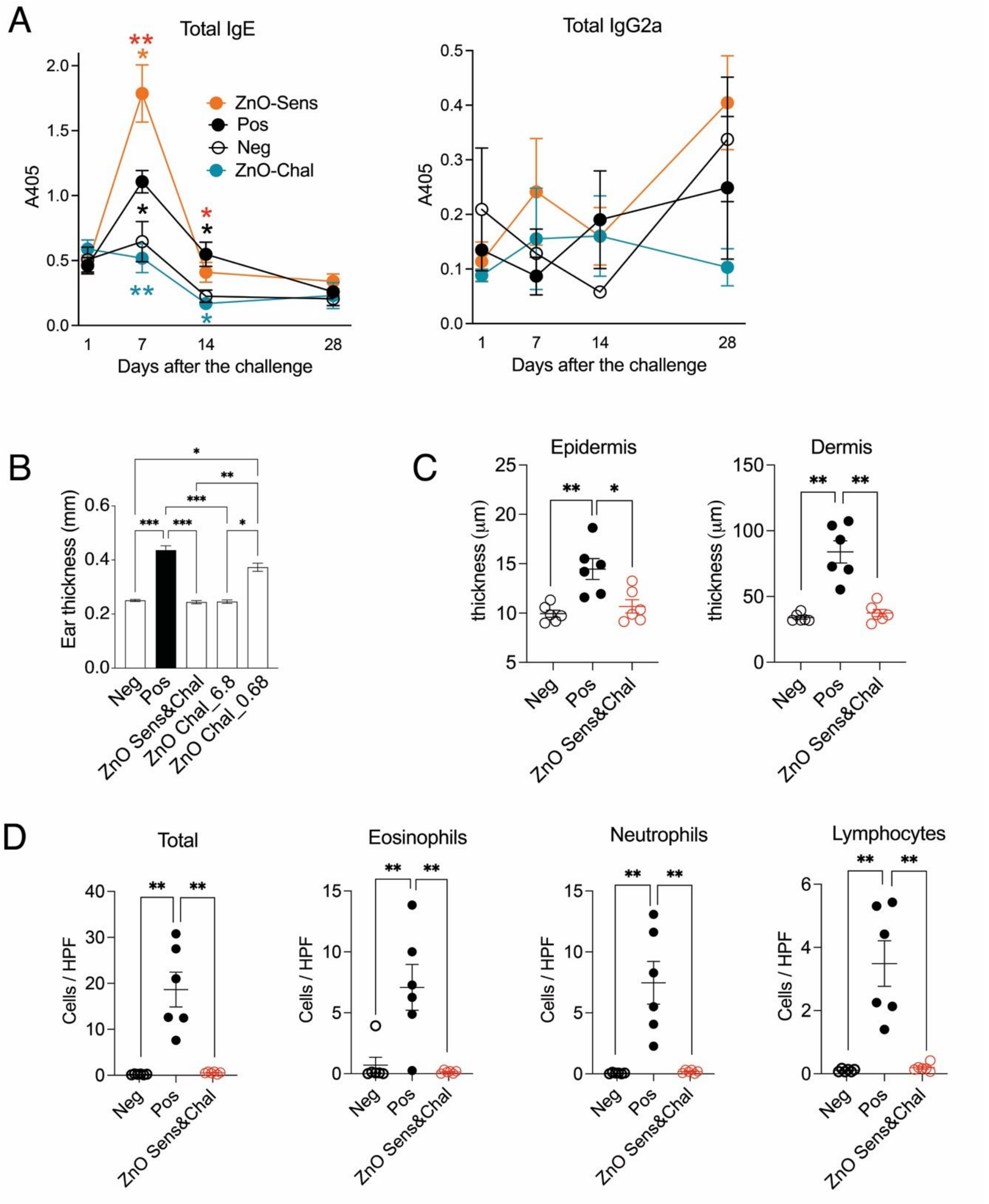

Figure S1. Effect of exposure to nZnO at both sensitization and challenge. A, Total IgE and IgG2a level changes in sera during 28 days of follow-up time are shown as optical values measured at 405 nm. Black stars represent comparison between Neg vs Pos, blue stars nZnO-challenge vs Pos, orange stars between nZnO-sensitization vs Pos, and red stars between nZnO-challenge vs nZnO-sensitization. Differences between the groups were studied by two-way ANOVA with Benjamini-Hochberg multiple comparison correction. B, Ear thickness was measured at sacrifice by a micrometer at 24 hours after the challenge. Differences between the groups (N=8/group) were studied by Kruskal–Wallis test with Dunn's multiple comparison correction, *P <0.05; **P <0.01; ***P <0.001; bars represent mean ±SEM. C, The thicknesses of ear dermis and epidermis layers were measured from H&E-stained ear sections under light microscope. D, Counting of eosinophils, neutrophils and lymphocytes and their summed total cells from the H&E-stained ear sections. Differences between the groups were studied by Mann–Whitney U-test. The scatter plots represent mean + SEM showing all datapoints.

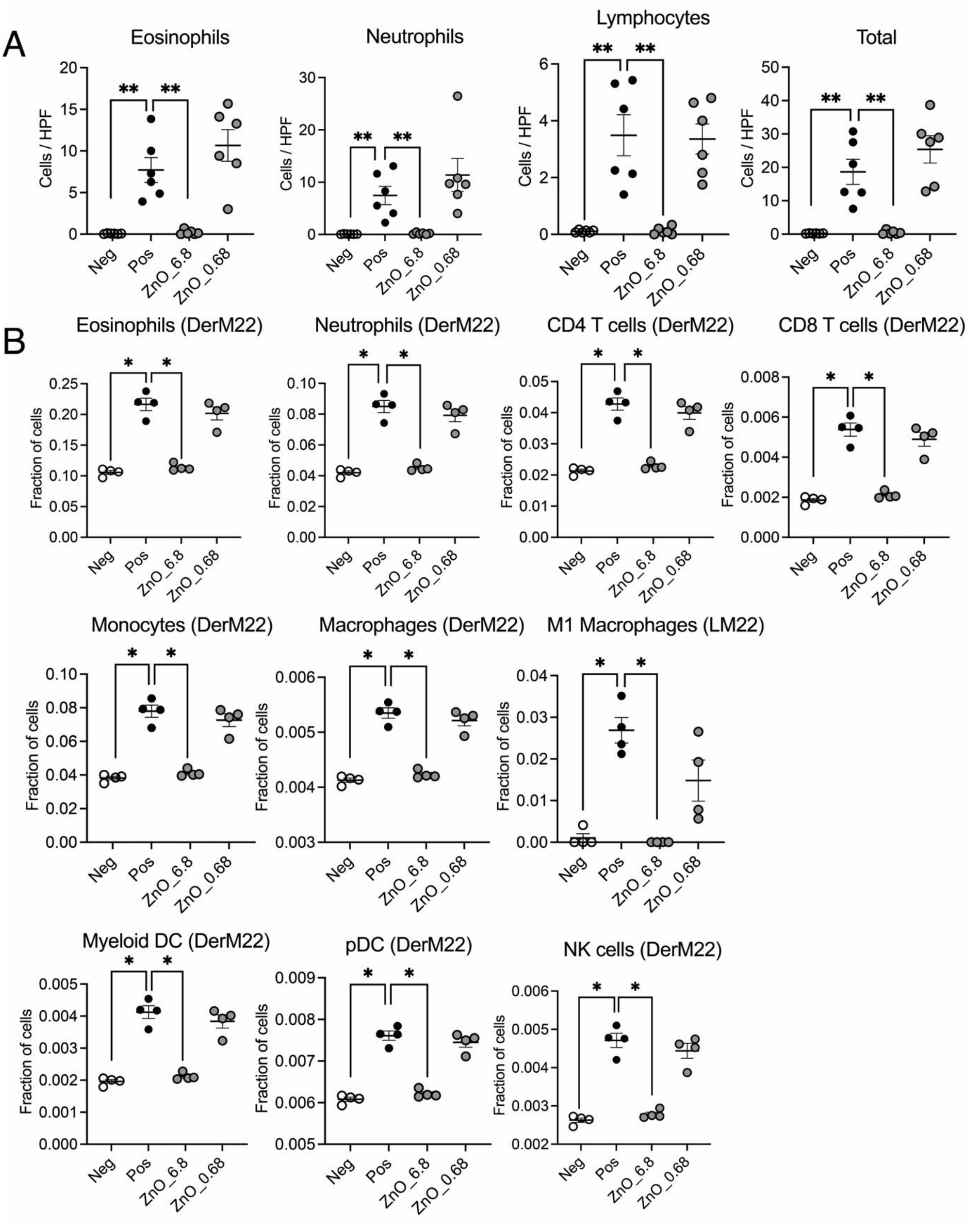

Figure S2. Effects of varied doses of nZnO on skin infiltrating immune cell profiles after 24 hours. A, The numbers of eosinophils, neutrophils and lymphocytes were counted from the H&E-stained ear tissue sections of negative, positive mice, and mice treated with 6.8 mg and 0.68 mg of nZnO at challenge. B, CIBERSORT-derived predication of immune cell subtype proportions using mouse orthologs-converted DerM22 and LM22 gene signature matrices. Differences between the groups were studied by by Mann–Whitney U-test, *P <0.05; **P <0.01. The scatter plots represent mean + SEM showing all datapoints.

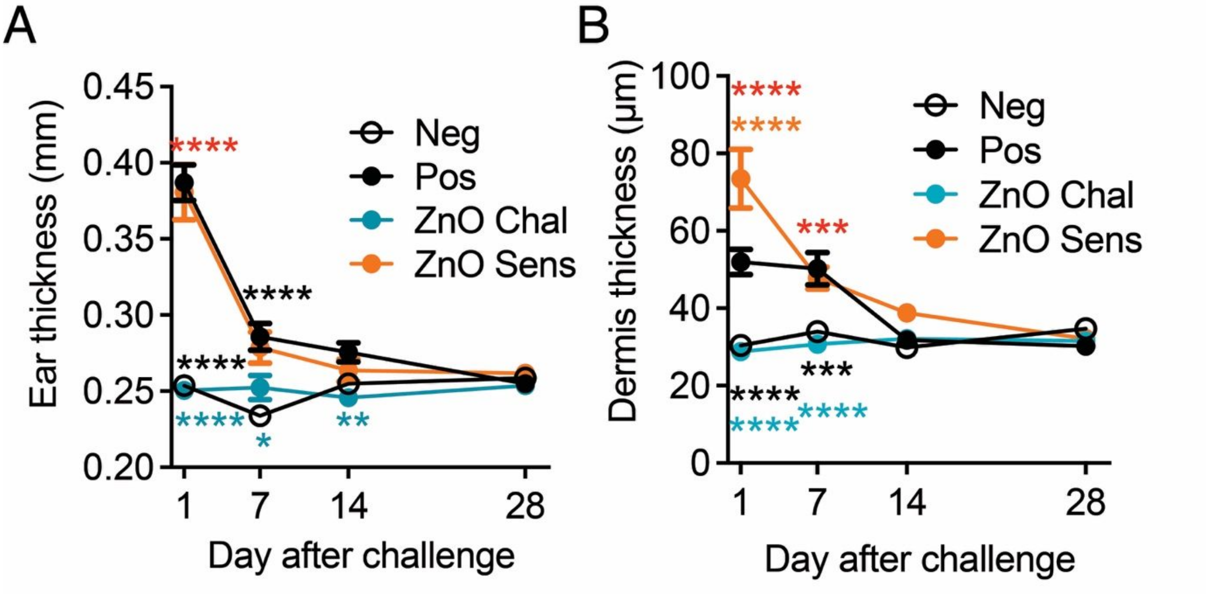

Figure S3. Effects of nZnO on skin thickness in CHS model during the 28 days of follow-up time. A, Whole ear thickness was measured just after the mouse sacrifice by micrometer at 1, 7, 14 or 28 days after the challenge. B, The thicknesses of ear dermis and epidermis were measured from the hematoxylin and eosin (H&E) -stained ear sections under light microscope. Differences between the groups were studied by two-way ANOVA with Tukey's multiple comparison correction, *P <0.05; **P <0.01; ***P <0.001; ****P <0.0001; bars represent mean ±SEM. Black stars represent comparison between Neg vs Pos, blue stars nZnO-challenge vs Pos, orange stars between nZnO-sensitization vs Pos, and red stars between nZnO-challenge vs nZnO-sensitization.

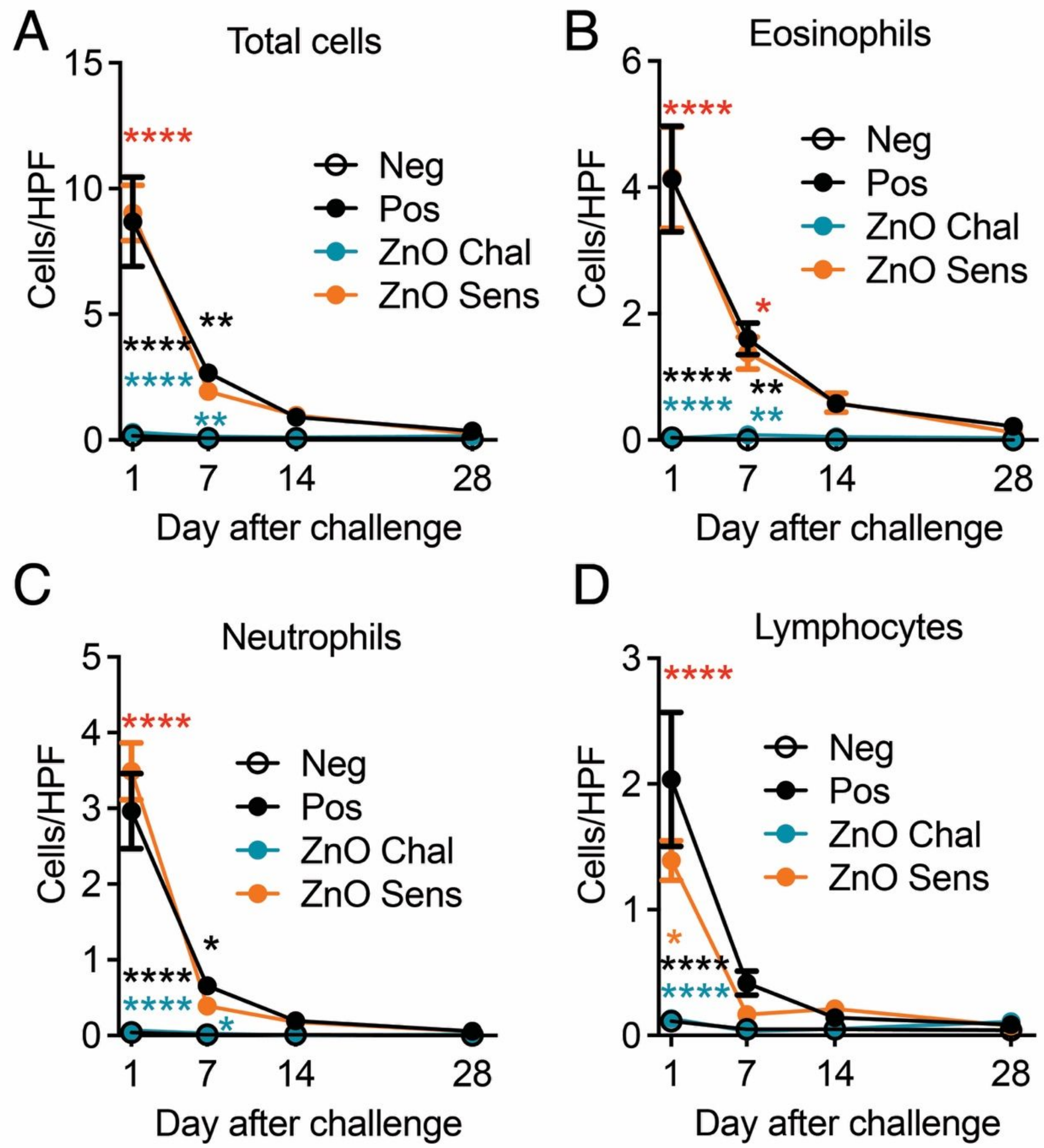

Figure S4. Effects of nZnO on cellular infiltration in CHS model during the 28 days of follow-up time. The number of A, total cells, B, eosinophils, C, neutrophils and D, lymphocytes were counted from the H&E-stained ear tissue sections under light microscope by 1000× magnification at 1, 7, 14 or 28 days after the challenge. Differences between the groups were studied by two-way ANOVA with Tukey's multiple comparison correction, *P <0.05; **P <0.01; ****P <0.0001; The bars represent mean ±SEM. Black stars represent comparison between Neg vs Pos, blue stars nZnO-challenge vs Pos, orange stars between nZnO-sensitization vs Pos, and red stars between nZnO-challenge vs nZnO-sensitization.

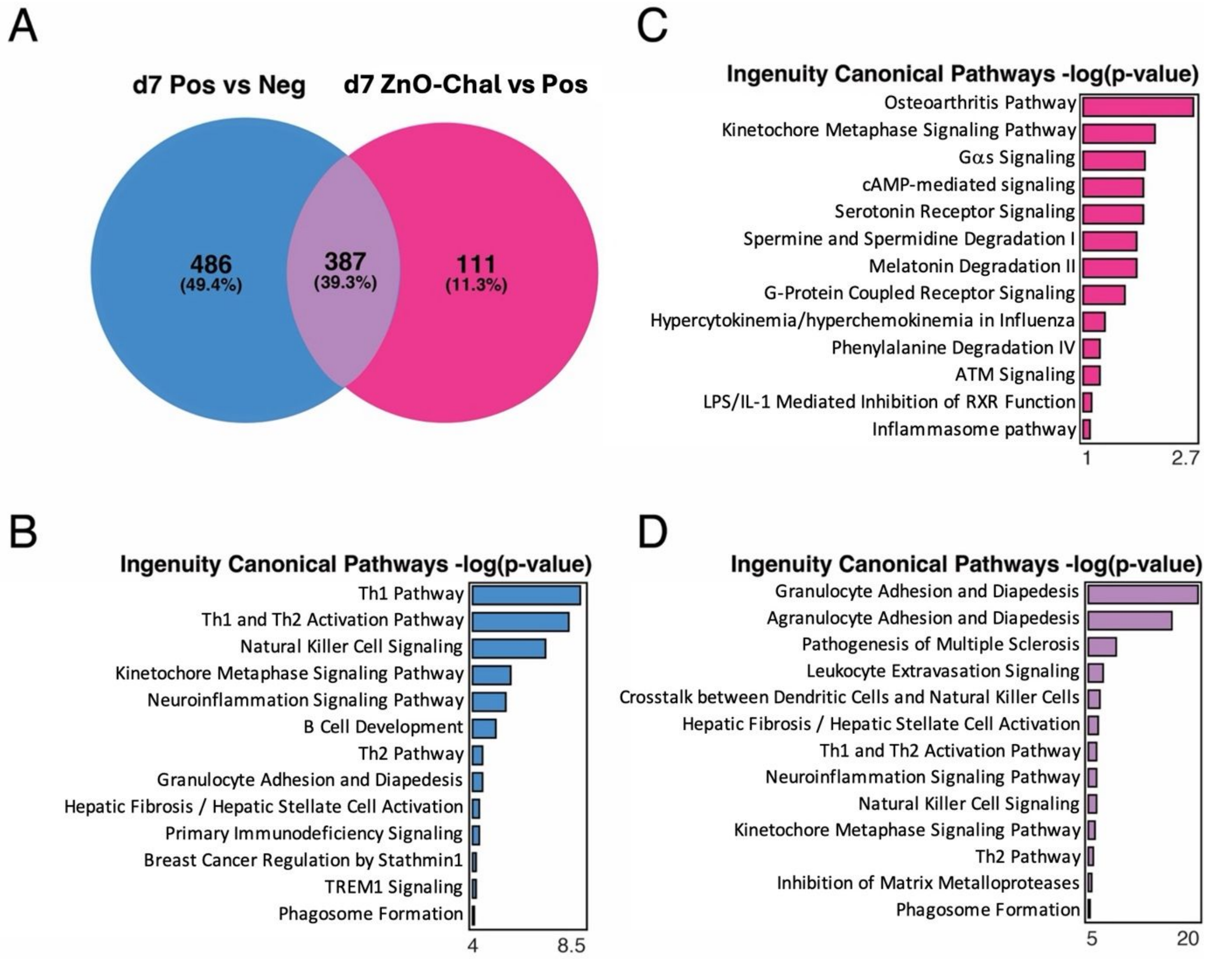

Figure S5. VENN-diagram of differentially expressed genes (DEGs) and associated pathway analyses of comparison-specific or shared DEGs at day 7. A, A VENN diagram shows DEGs are specific or shared between Pos vs Neg and ZnO-Chal vs Pos comparisons. The Ingenuity Canonical pathways were studied on B, 486 Pos vs Neg -specific DEGs, C, 111 ZnO-Chal vs Pos -specific DEGs and D, 387 shared DEGs within all groups. The negative logarithm of P-value from Fisher's exact test is shown for each enriched pathway.

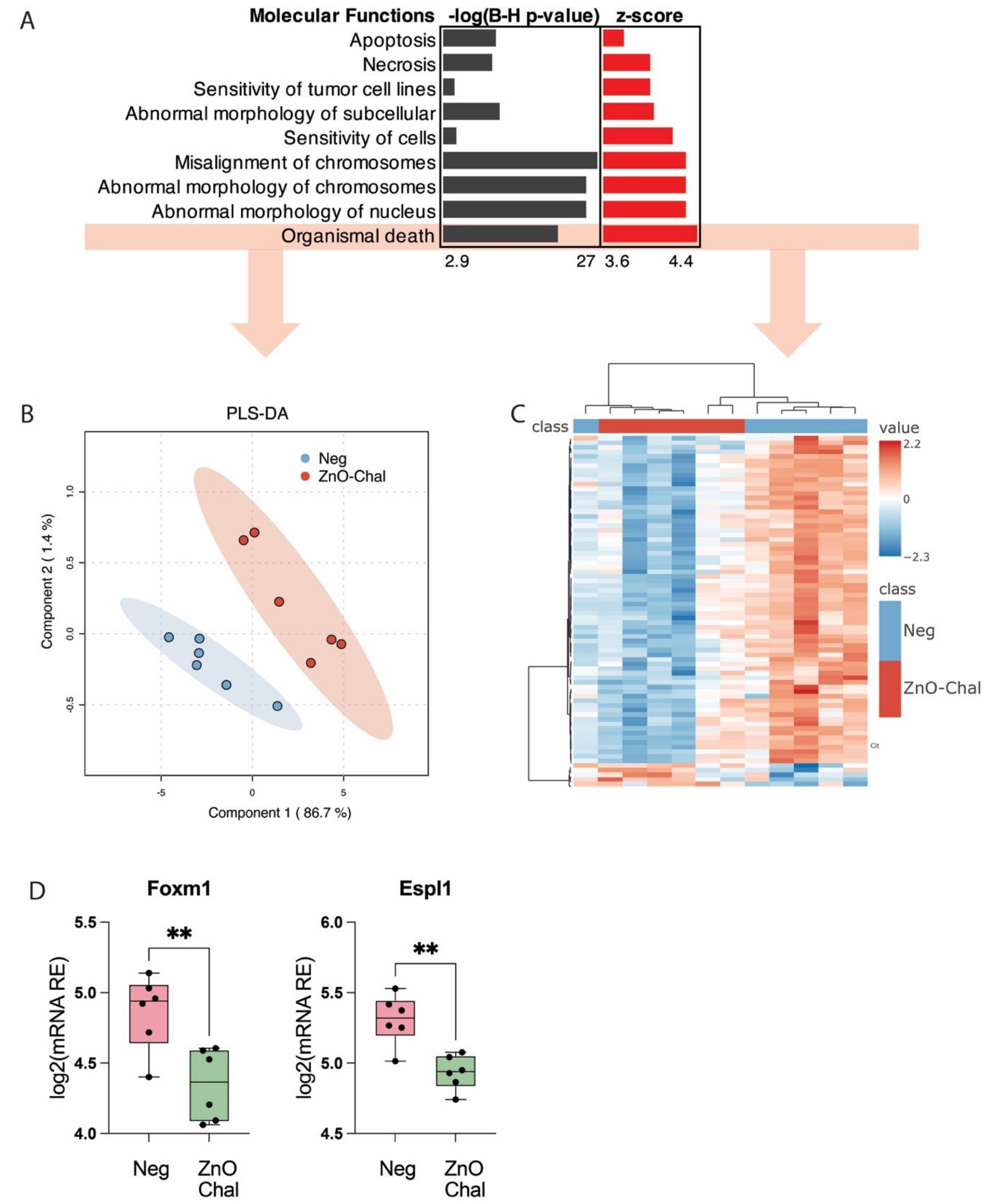

Figure S6. Cytotoxicity-related pathways in Module 4 of the ZnO-Chal vs. Neg comparison on day 1. A, Ingenuity Pathway Molecular Functions analysis. Genes related to the organismal death pathway were further characterized in panels B–D. B, Partial least squares-discriminant analysis (PLS-DA) of organismal death related genes reveals two distinct groups. C, Heatmap of ear skin gene expression in Module 4 by Euclidean clustering show that cytotoxicity-related cell survivability genes are downregulated in the ZnO-Chal group. D. Expression levels of Foxm1, a cell cycle gene involved in DNA replication and mitosis, and Espl1, a gene involved in chromosome segregation, are shown for each group. Differences between the groups were analyzed using an unpaired T-test, **P <0.01.

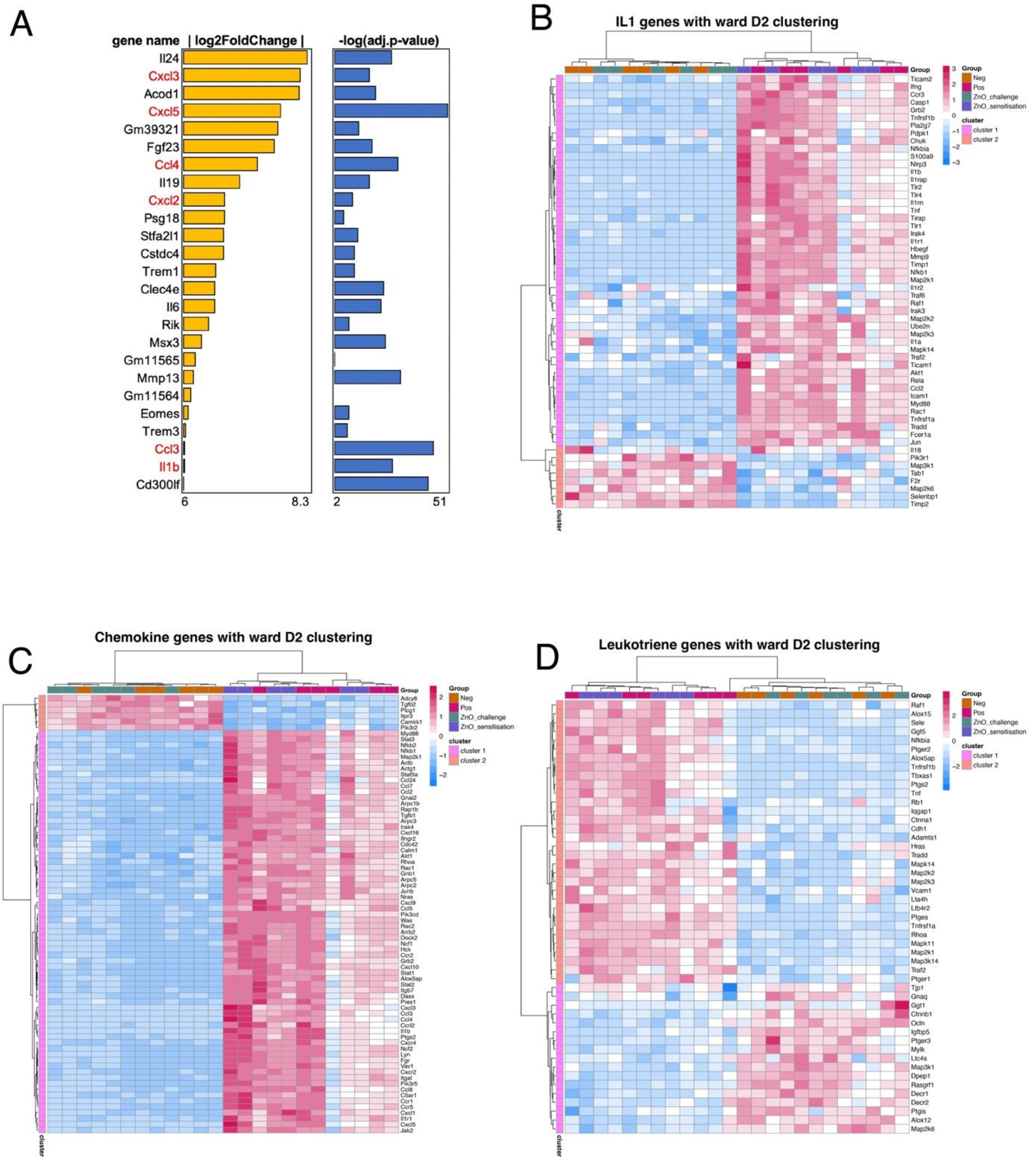

Figure S7. Involvement of DEGs associated with IL-1R, CXCR2 and LTB4 after 24 hours in CHS. A, The top 25 most differentially expressed genes found between ZnO-Chal vs Pos after 24 hours are shown with their absolute log2-fold change and their negative logarithm of Benjamini-Hochberg adjusted p-value. Clustering of Z-scored expression values of normalized counts of genes that are associated with terms B, "IL1", C, "chemokine" (top 75 genes are shown), and D, "leukotriene", based on term search function in Enrichr analysis tool.

Table S1. Particle characteristics provided by the manufacturer.

**Type**	**Nano**
**Size**	20 nm
**Form**	Particle
**Specific surface area**	50 m^2/g
**Purity**	99.50 %
**Coating**	None
**Dissolution**	Not provided

Table S2. Sizes of particle and their agglomerates studied by transmission electron microscopy.

	**Primary particle**	**Agglomerate**
**Size range (nm)**	5.0 –32.3	25.7–661.4
**Geometric mean diameter ± standard deviation (nm)**	15.6 ± 5.1	126.1±154.8
**Counts**	157	49
